# Prognostic value of preoperative P-CRP in patients with osteosarcoma: A retrospective study of 101 cases

**DOI:** 10.1097/MD.0000000000030382

**Published:** 2022-09-02

**Authors:** Hui Peng, Xu Fang, Yinglong Xu, Linhua Wei, Yiwu Qin, Fuchun Yang, Shenglin Lu, Jinmin Zhao

**Affiliations:** a Department of Orthopedics and Hand Surgery, The First Affiliated Hospital of Guangxi Medical University, Nanning, Guangxi, China; b Department of Orthopaedics, Minzu Hospital of Guangxi Zhuang Autonomous Region, Nanning, Guangxi, China; c Guangxi Key Laboratory of Regenerative Medicine, Research Centre for Regenerative Medicine, Guangxi Medical University, Nanning, Guangxi, China; d Department of Traumatology and Microsurgery, The First People’s Hospital of Nanning, Nanning, Guangxi, China.

**Keywords:** osteosarcoma, P-CRP, prognosis

## Abstract

This study aimed to investigate the value of the product of peripheral blood platelet and serum C-reactive protein (P-CRP), an inflammatory indicator, for the prognosis of patients with osteosarcoma. Patients with osteosarcoma who were diagnosed and treated at the First Affiliated Hospital of Guangxi Medical University, China, between January 2012 and December 2019 were included in this retrospective study. Receiver operating characteristic curves were used to calculate the optimal cut-off values for inflammatory indicators such as P-CRP, the C-reactive protein/albumin ratio (CRP/Alb), the neutrophil–lymphocyte ratio (NLR), and the platelet–lymphocyte ratio (PLR) in the peripheral blood of patients before treatment. Based on the cut-off values, the patients were divided into high P-CRP and low P-CRP groups, high CRP/Alb and low CRP/Alb groups, high NLR and low NLR groups, and high NLR and low NLR groups; the Kaplan–Meier method was used to compare the overall survival (OS) rates and OS times of the above groups. Univariate and multivariate Cox regression models were used to analyze the effects of various factors on the prognosis of osteosarcoma and to determine the independent influencing factors. The Kaplan–Meier survival analysis results suggested that the OS rate of the high P-CRP group was significantly lower than that of the low P-CRP group (14.0% vs 67.2%, *P* < .001). The univariate analysis results suggested that tumor volume, tumor stage, NLR, PLR, P-CRP and CRP/Alb were factors that affected the prognosis of patients with osteosarcoma, and the differences were statistically significant (*P* < .05). The multivariate analysis results showed that tumor volume (hazard ratio [HR] = 1.061; 95% CI, 1.001–1.125; *P* = .046) and preoperative P-CRP (HR, 1.037; 95% CI, 1.024–1.050; *P* < .01) were independent prognostic factors affecting the OS rate after osteosarcoma surgery. The results of our study showed that P-CRP is a novel and promising prognostic indicator for patients with osteosarcoma. The higher the P-CRP level in the peripheral blood of patients is before treatment, the worse the prognosis might be.

## 1. Introduction

Osteosarcoma, which occurs primarily in children and adolescents, has an annual incidence of approximately 4.4/1,000,000, and accounts for approximately 45% of all bone tumors.^[1]^ Osteosarcoma has a high degree of malignancy, and pulmonary metastasis can occur at an early stage. Therefore, the prognosis of this disease is extremely poor. The traditional factors that affect the prognosis of osteosarcoma include tumor grade, histological subtype, and metastasis.^[2,3]^ However, it is difficult to accurately and comprehensively predict the prognosis of osteosarcoma using these factors alone. Therefore, the search for simple, novel, and effective predictors is of great significance.

The systemic inflammatory response has been recognized as the seventh major hallmark of cancers.^[4,5]^ C-reactive protein (CRP) is a serum-based marker of the systemic inflammatory response and is a predictor of low survival rates for patients with cervical cancer and pancreatic cancer. The product of peripheral blood platelet and serum C-reactive protein (P-CRP) sensitively and accurately predicts the prognosis of malignant tumors by fully combining the characteristics of PLT and CRP elevation that result in a poor cancer prognosis.^[6–9]^

To our knowledge, there is no report on the prognostic value of P-CRP in patients with osteosarcoma. Therefore, this retrospective study investigated the impact of preoperative serum P-CRP values on the survival prognosis of patients with osteosarcoma.

## 2. Patients and methods

### 2.1. Sample size estimation

In order to perform the Cox regression for multivariate analysis scientifically as well as feasible, we included patients 13 times variables as study size.

### 2.2. Inclusion and exclusion criteria

Patients with osteosarcoma diagnosed and treated at the First Affiliated Hospital of Guangxi Medical University, China, between January 2012 and December 2019 were included in this retrospective study. The inclusion criteria were as follows: postoperative histopathological diagnosis of osteosarcoma; no anticancer treatment, including chemotherapy, radiotherapy, or blood transfusion, before admission; no other systemic comorbidities or underlying diseases that affected the patient’s prognosis; serum CRP, platelet counts, and albumin levels available at the time of diagnosis; sufficient clinicopathological and follow-up data with detailed medical history and examination results. The exclusion criteria were as follows: preoperative infection, high fever, autoimmune diseases, or hematological diseases; other organ dysfunction; history of other malignant tumors; the use of non-steroidal anti-inflammatory drugs or antibiotics; and loss to follow-up or death due to non-cancer causes.

### 2.3. Data collection

The clinical data of the patients, including the patient’s sex, age, tumor size, Enneking stage, neoadjuvant chemotherapy, body mass index, and other basic clinical information, such as laboratory test results, were retrieved and collected through the hospital information system. The absolute neutrophil, lymphocyte, and platelet counts and levels of CRP and Alb were obtained from fasting anterior cubital venous blood samples taken after admission and before intervention. P-CRP (primary variable) was calculated by multiplying the peripheral platelet count by the serum CRP value and then dividing by 1000. The neutrophil–lymphocyte ratio (NLR) is the neutrophil count divided by the lymphocyte count. The platelet–lymphocyte ratio (PLR) is the platelet count divided by the lymphocyte count. C-reactive protein/albumin ratio (CRP/Alb) was calculated by dividing the serum CRP value by the serum Alb value. All of these data were independently collected by 3 clinicians and then verified and recorded in EXCEL to reduce bias in data collection.

### 2.4. Follow-up

The patients were followed regularly through outpatient follow-up visits, phone calls, and WeChat. Overall survival (OS) time was defined in this study as the period from the time of histopathological confirmation of the diagnosis to the date of the last follow-up or death. Follow-up was conducted once every 3 months for the first year and then once every 6 months until the most recent follow-up. The contents of the follow-up included a comprehensive medical history, physical examination, laboratory examination, computed tomography, and other items.

### 2.5. Ethics approval

All research procedures used in this study were approved by the Ethics Committee of the First Affiliated Hospital of Guangxi Medical University. Reference number: NO.2022-KY-E-(093).

### 2.6. Statistical analysis

Statistical software SPSS 24.0 was used for data analysis. The chi-square test was used for comparisons among groups. The receiver operating characteristic curve was plotted, and the Youden index (sensitivity + specificity -1) was used to determine the optimal cut-off values for NLR, PLR, CRP/Alb, and P-CRP. The patients were divided into groups (high or low P-CRP, high or low NLR, high or low PLR, and high or low CRP/Alb) based on the optimal cut-off values. The Kaplan–Meier method was used to plot the survival curve, and the log-rank test was used to compare the difference in survival between the 2 groups. A Cox proportional hazards regression model was used for univariate and multivariate analysis of potential predictive factors to determine which factors were independent predictors of the OS for osteosarcoma. *P* < .05 was considered statistically significant.

## 3. Results

### 3.1. Analysis of basic patient data

A total of 101 patients were enrolled in this study, including 67 males (66.3%) and 34 females (33.7%). The median age was 18 years, and most of the patients had tumors in the limb bones (86.1%). There were 19, 43, and 39 cases of stage I, II, and III tumors, respectively, accounting for 18.8%, 42.6%, and 38.6% of the patients, respectively. Twenty-one patients (20.8%) received neoadjuvant chemotherapy (Table 1).

**Table 1:** Clinicopathological characteristics of patients.

	Patients	Low P-CRP	High P-CRP	*P* value
Total	101	58	43	-
Gender				.139
Male	67	35	32	
Female	34	23	11	
Age (yr)				.326
<18	48	30	18	
≥18	53	28	25	
BMI	18.78 ± 3.82	18.16 ± 3.57	19.63 ± 4.03	.054
Tumor location				.320
Extremities	87	48	39	
Others	14	10	4	
Size (cm)	8.05 ± 4.78	7.01 ± 3.96	9.44 ± 5.45	.015
Enneking stage				.001
I + II	62	42	20	
III	39	16	23	
NACT				.976
Yes	21	12	9	
No	80	46	34	
NLR				.018
Low	49	34	15	
High	52	24	28	
PLR				.023
Low	31	23	8	
High	70	35	35	
CRP/ALB				<.001
Low	23	22	1	
High	78	36	42	
ALP				.084
Low	50	33	17	
High	51	25	26	
Surgical method				.230
Limb salvage	26	15	11	
amputation	75	30	25	

ALP = alkaline phosphatase, BMI = body mass index, CRP/ALB = C-reactive protein to albumin ratio, NACT = neoadjuvant chemotherapy, NLR = neutrophil–lymphocyte, P-CRP = platelet and serum C-reactive protein, PLR = platelet–lymphocyte ratio.

### 3.2. Optimal cut-off values for each indicator

**Figure 1.**
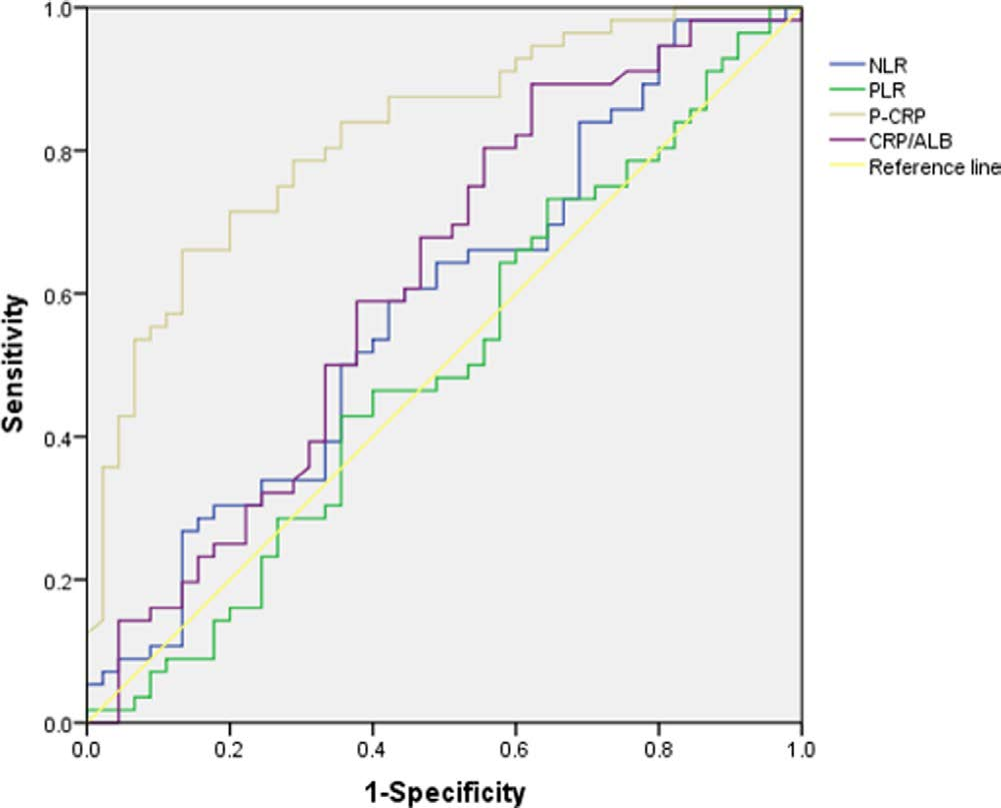
The receiver operating characteristic curves (ROC) among the inflammation-based prognostic indicators.

As shown in Figure 1 and Table 2, the optimal cut-off values for P-CRP, NLR, PLR, and CRP/ALB were 3.904, 2.096, 105.17, and 0.05, respectively, and the areas under the curve were 82.3%, 58.3%, 50.0%, and 61.7%, respectively. According to the optimal cut-off values, the patients were divided into a high P-CRP group (HP-CRP ≥ 3.904, n = 43) and a low P-CRP group (<3.904, n = 58), a high NLR group (HNLR ≥ 2.096, n = 52) and a low NLR group (LNLR < 2.096, n = 49), a high PLR group (HPLR ≥ 105.17, n = 70) and a low PLR group (LPLR < 105.17, n = 31), and a high CRP/Alb group (≥0.05, n = 78) and a low CRP/Alb group (<0.05, n = 23).

**Table 2:** Comparison of the AUCs and cutoff value between inflammation-based prognostic indicator.

	AUC	SE	*P*	95% CI	Cutoff value
NLR	0.583	0.058	.155	0.470–0.695	2.096
PLR	0.500	0.059	.995	0.385–0.616	105.173
P-CRP	0.823	0.041	<.001	0.742–0.903	3.904
CRP/ALB	0.617	0.057	.043	0.505–0.730	0.050

AUC = area under the curve, CRP/ALB = C-reactive protein to albumin ratio, NLR = neutrophil–lymphocyte, P-CRP = platelet and serum C-reactive protein, PLR = platelet–lymphocyte ratio.

### 3.3. Relationship between P-CRP and clinicopathological data

The relationship between P-CRP and various clinical characteristics of the patients with osteosarcoma is shown in Table 1. Compared with the high P-CRP group, the low P-CRP group had a smaller tumor volume (7.01 ± 3.96 cm vs 9.44 ± 5.45 cm, *P* = .015); similarly, the tumor stage tended to be earlier (stage I + II) in the low P-CRP group than in the high P-CRP group (72.4% vs 46.5%, *P* = .001). However, there was no significant difference between the 2 groups in terms of age, sex, tumor site, body mass index, neoadjuvant chemotherapy or surgical method (*P* > .05).

### 3.4. Relationship between inflammatory indicators and survival rate

**Figure 2.**
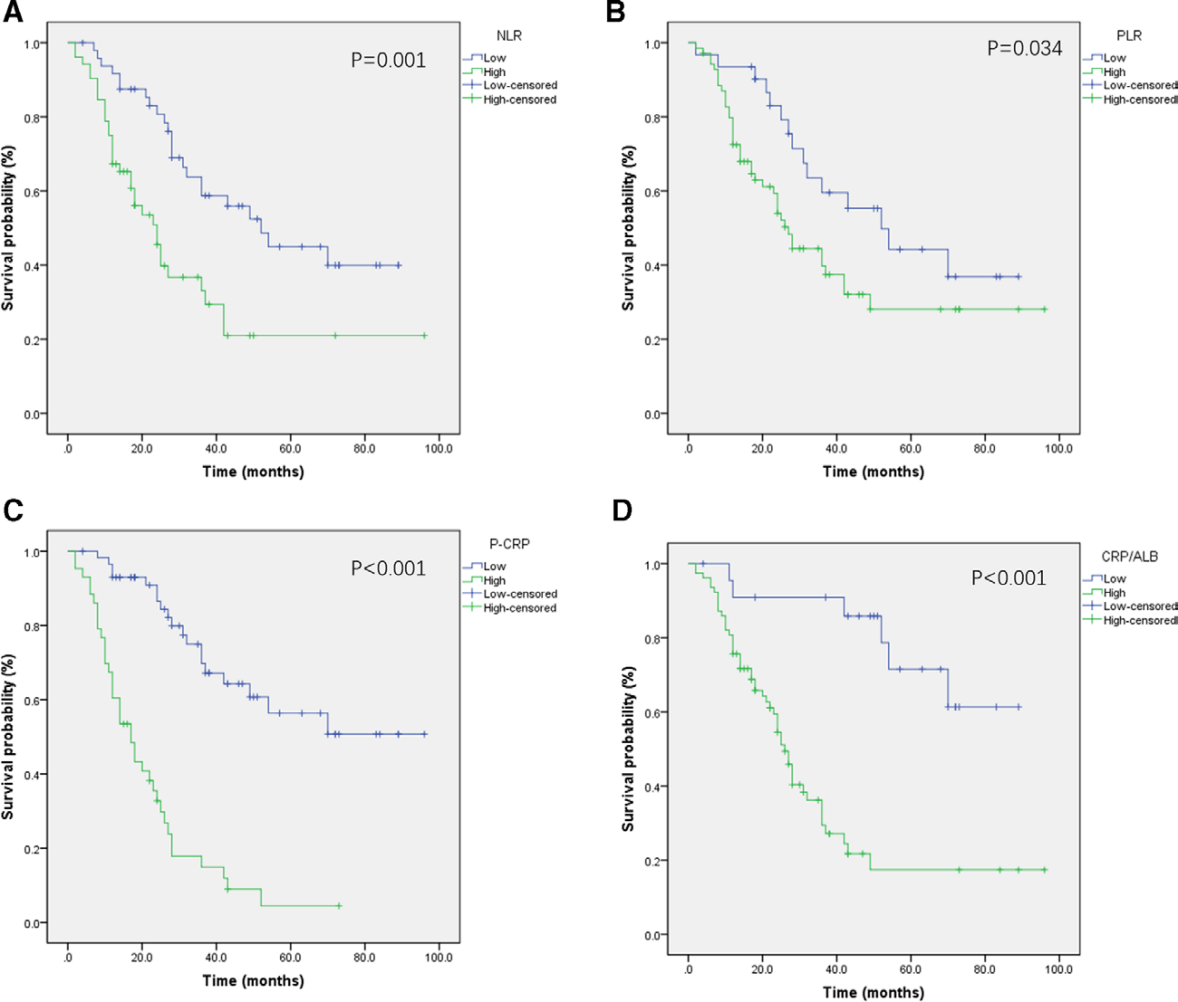
Kaplan–Meier survival curves for overall survival in our osteosarcoma patients according to NLR, PLR, P-CRP and CRP/ALB. CRP/Alb = C-reactive protein/albumin ratio, NLR = neutrophil–lymphocyte ratio, P-CRP = platelet and serum C-reactive protein, PLR = platelet–lymphocyte ratio.

The OS rate of all patients was 44.6%, and the median OS time was 30.88 ± 22.38 months. As shown in Figure 2, the OS rates of patients in the different P-CRP groups were compared, and the survival curves suggest that the OS rate of the high P-CRP group were lower than that of the low P-CRP group, with a statistically significant difference (14.0% vs 67.2%, *P* < .001). Similarly, the OS rate of the high NLR group was lower than of the low NLR group (36.5% vs 53.1%, *P* = .001), the OS rate of the high PLR group was lower than of the low PLR group (41.4% vs 51.6%, *P* = .034), and the OS rate of the high CRP/Alb group was lower than that of the low CRP/Alb group (35.6% vs 73.9%, *P* < .001).

### 3.5. Univariate and multivariate analysis

The univariate analysis of potential factors affecting the prognosis of osteosarcoma patients showed that tumor volume, tumor stage, surgical method, P-CRP, NLR, and CRP/Alb affected the prognosis of osteosarcoma patients, and the differences were statistically significant (*P* < .05) (Table 3). These factors were included in the Cox regression for multivariate analysis. The results showed that tumor volume (hazard ratio [HR] = 1.061; 95% confidence interval (CI), 1.001–1.125; *P* = .046) and preoperative P-CRP (HR, 1.037; 95% CI, 1.024–1.050; *P* < .01) were independent prognostic factors affecting the postoperative OS of osteosarcoma patients (Table 3).

**Table 3:** Univariate and multivariate analyses of overall survival using the cox proportional hazard model.

	Univariate analysis			Multivariate analysis		
	*P*	HR	95% CI	*P*	HR	95% CI
Gender	.325	0.747	0.417–1.336			
Age	.437	1.007	0.990–1.025			
Location	0.638	0.959	0.806–1.142			
Size	**.004**	**1.080**	**1.025–1.137**	**.046**	**1.061**	**1.001–1.125**
Enneking stage	**<.001**	**2.999**	**1.977–4.548**			
NACT	.438	0.776	0.408–1.474			
BMI	.257	1.037	0.974–1.103			
NLR	**.029**	**1.137**	**1.013–1.275**	.113	1.153	0.967–1.374
PLR	.354	1.002	0.998–1.005			
P-CRP	**<.001**	**1.039**	**1.028–1.050**	**<.001**	**1.037**	**1.024–1.050**
CRP/ALB	**.004**	**1.232**	**1.067–1.421**	.773	0.973	0.810–1.170
ALP	.137	1.000	1.000–1.001			
Surgical method	**.043**	**1.145**	**1.894–1.257**	.376	0.983	0.996–1.007

ALK = alkaline phosphatase, BMI = body mass index, CRP/ALB = C-reactive protein to albumin ratio, NACT = neoadjuvant chemotherapy, NLR = neutrophil–lymphocyte, P-CRP = platelet and serum C-reactive protein, PLR = platelet–lymphocyte ratio.

## 4. Discussion

This study conducted a series of analyses of the clinicopathological data, inflammatory indicator data, and prognosis follow-up data of 101 patients with osteosarcoma. We identified and proved the value of P-CRP as a novel inflammatory factor to predict the prognosis of osteosarcoma patients.

P-CRP was regarded as a valuable prognostic factor in many cancers. P-CRP has increased sensitivity and accuracy for predicting the prognosis of malignant tumors because it fully combining the characteristics of PLT and CRP elevation that result in a poor prognosis. A study by Ide S et al^[10]^ included 135 patients with locally advanced rectal cancer; the optimal cut-off value was 4.11, the survival curves showed that the OS of the high P-CRP group was lower than that of the low P-CRP group, and P-CRP was determined to be an independent factor affecting the prognosis of locally advanced rectal cancer patients. Yuji Shishido et al^[11]^ retrospectively analyzed 116 esophageal squamous cell carcinoma patients using an optimal cut-off value of 1.674. The results showed poorer OS in the high P-CRP group than in the low P-CRP group (46.4% vs 77.3%, *P* = .0056). Similar to these results, our study confirmed that P-CRP could predict the prognosis of patients with osteosarcoma. We enrolled 101 patients with osteosarcoma and calculated the optimal cut-off value using the receiver operating characteristic curve, which was 3.904. The results showed that the OS rate of the high P-CRP group was lower than that that of the low P-CRP group, and the difference was statistically significant (14.0% vs 67.2%, *P* < .001). The univariate and multivariate Cox regression analysis indicated that P-CRP could be used as an independent factor to predict the prognosis of osteosarcoma patients.

The reason for our finding can be explained as follows. CRP is synthesized by liver cells, lymphocytes, and smooth muscle cells. It plays an important role in the production of cytokines and is regulated by proinflammatory cytokines and interleukin-6 (IL-6). It is a common serum marker for the assessment of inflammatory status.^[12]^ Increased serum vascular growth factor and circulating interleukins in cancer patients contribute to elevated CRP and accelerated angiogenesis, which lead to drug resistance and low survival rates in patients with malignancies; thus, CRP has been shown to be a prognostic indicator for malignancies such as ovarian cancer, colorectal cancer, and oral cancer.^[13,14]^ Tumor cells stimulate platelet activation, and IL-6 induces the differentiation of megakaryocytes into platelets. In addition, the secretion of vascular endothelial growth factor in tumor cells increases, and these factors all result in an increase in reactive platelets. Thrombocytosis enhances the adhesion and infiltration of malignant tumor cells through the endothelial system, thus preventing the immune system from removing tumor cells from the circulatory system, where derived endothelial growth factors have mitogenic and angiogenic activity that promote tumor progression.^[15,16]^ In fact, the value of platelets as a predictor of prognosis in cases of malignant tumors has been reported in many studies.^[17]^

In addition, this study analyzed the prognostic value of other inflammatory factors in osteosarcoma. NLR and PLR are important indicators of the systemic inflammatory response in the body. Many studies have reported the relationship between these 2 indicators and the prognosis of malignant tumors. In a retrospective analysis conducted by Xia et al,^[18]^ 359 osteosarcoma patients were included. The results showed that NLR and PLR were important prognostic indicators for osteosarcoma patients and that the OS rates of patients with high NLR or high PLR were significantly worse than those of patients with low NLR or low PLR. Our data also showed that the OS of the high NLR group was lower than that of the low NLR group (36.5% vs 53.1%, *P* = .001) and that the OS of the high PLR group was lower than that that of the low PLR group (41.4% vs 51.6%, *P* = .034). Univariate and multivariate regression analysis showed that NLR and PLR no longer affected the prognosis of patients with osteosarcoma, which may be related to our small sample size.

CRP/Alb, obtained from the combination of CRP and albumin levels, can reflect the inflammation and nutritional status of cancer patients and provide a fully integrated view of the effects of inflammation and low nutritional status on the prognosis of cancer patients. The effect of CRP/Alb on the prognosis of osteosarcoma has been reported in the literature. Li et al^[19]^ analyzed 216 osteosarcoma patients and found that the OS rate of the high CRP/Alb group (>0.210) was significantly lower than that of the low CRP/Alb group (<0.210) and that CRP/Alb was an independent factor affecting the prognosis of patients with osteosarcoma (HR = 2.21, 95% CI = 1.40–3.49; *P* = .001). In our study, although CRP/Alb was not an independent factor affecting the OS rate of osteosarcoma patients, both univariate and survival curve analyses suggested that high CRP/Alb is indicative of a poor prognosis for osteosarcoma; therefore, there is still reason to believe that CRP/Alb is a potentially useful indicator for predicting the survival rate in osteosarcoma.

As we all know, the surgical method is one of the important ways to affect the prognosis of osteosarcoma. The treatment methods included limb salvage and amputation. Twenty-six and 75 patients underwent limb salvage and amputation, respectively. After lesion resections, patients would underwent different reconstructions methods. In general, custom-made tumor prostheses will be used when the lesion invaded the joints, while autologous fibula transplantation and the devitalization and replantation of tumor segment were applied for reconstruct of diaphysis lesion. Although the survival rate of amputation was higher than that of limb salvage group in univariate analysis, this difference was no longer significant in multivariate analysis. The reason for this phenomenon may be attributed to the insufficient sample size of this study.

## 5. Limitations

Firstly, some biases cannot be avoided because this is a retrospective study. What’s more, the sample size involved in this study is insufficient.

## 6. Conclusion

Our results showed that P-CRP is a novel and promising prognostic indicator for patients with osteosarcoma. The higher the P-CRP level in the peripheral blood of patients is before treatment, the worse the prognosis might be.

## Acknowledgments

The authors thank all the staff of the participating departments.

## Author contributions

**Conceptualization:** Hui Peng, Yiwu Qin, Fuchun Yang, Shenglin Lu, Jinmin Zhao.

**Data curation:** Hui Peng, Xu Fang, Yinglong Xu, Jinmin Zhao.

**Formal analysis:** Hui Peng, Yinglong Xu, Jinmin Zhao.

**Methodology:** Jinmin Zhao.

**Project administration:** Jinmin Zhao.

**Resources:** Jinmin Zhao.

**Software:** Yinglong Xu, Jinmin Zhao.

**Supervision:** Shenglin Lu, Jinmin Zhao.

**Validation:** Jinmin Zhao.

**Writing – original draft:** Hui Peng, Xu Fang, Linhua Wei, Fuchun Yang, Shenglin Lu, Jinmin Zhao.

**Writing – review & editing:** Yiwu Qin, Shenglin Lu, Jinmin Zhao.
